# Secretome screening reveals immunomodulating functions of IFNα-7, PAP and GDF-7 on regulatory T-cells

**DOI:** 10.1038/s41598-021-96184-z

**Published:** 2021-08-18

**Authors:** Mei Ding, Rajneesh Malhotra, Tomas Ottosson, Magnus Lundqvist, Aman Mebrahtu, Johan Brengdahl, Ulf Gehrmann, Elisabeth Bäck, Douglas Ross-Thriepland, Ida Isaksson, Björn Magnusson, Kris F. Sachsenmeier, Hanna Tegel, Sophia Hober, Mathias Uhlén, Lorenz M. Mayr, Rick Davies, Johan Rockberg, Lovisa Holmberg Schiavone

**Affiliations:** 1 Discovery Biology, Discovery Sciences, R&D, AstraZeneca, Gothenburg, Sweden; 2 Translational Science and Experimental Medicine, Research and Early Development, Respiratory and Immunology (R&I), BioPharmaceuticals R&D, AstraZeneca, Gothenburg, Sweden; 3 Department of Protein Science, School of Engineering Sciences in Chemistry, Biotechnology and Health, KTH - Royal Institute of Technology, Stockholm, Sweden; 4 Mechanistic Biology and Profiling, Discovery Sciences, R&D, AstraZeneca, Gothenburg, Sweden; 5 Discovery Biology, Discovery Sciences, R&D, AstraZeneca, Cambridge, UK; 6 Sample Management, Discovery Sciences, R&D, AstraZeneca, Gothenburg, Sweden; 7 Translational Medicine, Oncology R&D, AstraZeneca, Boston, USA

**Keywords:** Target identification, Immunology

## Abstract

Regulatory T cells (Tregs) are the key cells regulating peripheral autoreactive T lymphocytes. Tregs exert their function by suppressing effector T cells. Tregs have been shown to play essential roles in the control of a variety of physiological and pathological immune responses. However, Tregs are unstable and can lose the expression of FOXP3 and suppressive functions as a consequence of outer stimuli. Available literature suggests that secreted proteins regulate Treg functional states, such as differentiation, proliferation and suppressive function. Identification of secreted proteins that affect Treg cell function are highly interesting for both therapeutic and diagnostic purposes in either hyperactive or immunosuppressed populations. Here, we report a phenotypic screening of a human secretome library in human Treg cells utilising a high throughput flow cytometry technology. Screening a library of 575 secreted proteins allowed us to identify proteins stabilising or destabilising the Treg phenotype as suggested by changes in expression of Treg marker proteins FOXP3 and/or CTLA4. Four proteins including GDF-7, IL-10, PAP and IFNα-7 were identified as positive regulators that increased FOXP3 and/or CTLA4 expression. PAP is a phosphatase. A catalytic-dead version of the protein did not induce an increase in FOXP3 expression. Ten interferon proteins were identified as negative regulators that reduced the expression of both CTLA4 and FOXP3, without affecting cell viability. A transcriptomics analysis supported the differential effect on Tregs of IFNα-7 versus other IFNα proteins, indicating differences in JAK/STAT signaling. A conformational model experiment confirmed a tenfold reduction in IFNAR-mediated ISG transcription for IFNα-7 compared to IFNα-10. This further strengthened the theory of a shift in downstream messaging upon external stimulation. As a summary, we have identified four positive regulators of FOXP3 and/or CTLA4 expression. Further exploration of these Treg modulators and their method of action has the potential to aid the discovery of novel therapies for both autoimmune and infectious diseases as well as for cancer.

## Introduction

Regulatory T cells (Tregs) are key cells in the immune system. Tregs suppress effector T cells by regulating peripheral autoreactive T lymphocytes. Tregs have been shown to play essential roles in the control of a variety of physiological and pathological immune responses including inflammatory conditions, autoimmune diseases, and cancer^1–3^. A number of human autoimmune diseases, including systemic lupus erythematosus (SLE) and rheumatoid arthritis (RA), have been shown to be caused by disruption of Treg cell function or development of Tregs^3–5^.

There are two types of Treg cells in vivo: naturally occurring Tregs that are antigen-primed and generated in the thymus, and peripheral/induced Tregs that are differentiated from naïve conventional T cells (Tconv). Both natural and induced Tregs exert suppressive activity and express the transcription factor forkhead box p3 (FOXP3), recognised as the ‘master regulator’ of Treg cell function^2^. Recent studies have shown that peripheral and induced Treg cells are unstable and can lose FOXP3 expression and suppressive function^6^. Treg cell function and FOXP3 expression play important roles in autoimmune and inflammatory diseases. New modulators regulating FOXP3 expression and its downstream genes will aid the discovery of novel signaling pathways and targets affecting Treg cell function and stability. One downstream gene is cytotoxic T-lymphocyte antigen-4 (CTLA4).Treg cells produce and secrete many proteins including IL-10, TGFβ, IL35, galectin-1^7–10^ to exert the suppressive function on T effector cells. A vast number of receptors including CD3, CD4, CD25, GITR (glucocorticoid-induced tumour necrosis factor receptor), CD49b, type 1 IFNα receptor^5,11–14^ have also been shown to be expressed on the cell surface of Treg cells. Numerous studies have demonstrated that secreted factors are involved in regulating Treg cell differentiation, proliferation and suppressive function. Chen et al. demonstrated that IL2 and TGFβ were essential for differentiating naïve CD4+ cells to Treg cells^15–17^. Several studies showed that IL-10 potentiates Treg differentiation and maintains FOXP3 expression^18^, and type-1 interferon negatively regulates FOXP3 expression in Treg cells^19^. These results highlight that the secretome, consisting of proteins actively being secreted from cells^20^, is of importance for Treg cell function. Thus, screening for effect of the secretome on Treg cells would provide an opportunity to identify relevant protein signals that regulate Treg cell function and stability, and improve our understanding of the role of the secretome in Treg cell function.

We have recently developed an assay which measures the stability of the Treg protein markers FOXP3 and CTLA4 in primary human Tregs. We have applied this automated high-throughput assay to identify potent and novel small molecule regulators of the Treg markers and as indicators of the Treg phenotype^21^, using a diverse small compound library. This resulted in the discovery of intracellular proteins euchromatic histone-lysine N-methyltransferase 2 (EHMT2) and glycogen synthase kinase 3 alpha/beta (GSK3α/*β*) as positive FOXP3 regulators, and bromodomain and extraterminal domain (BET) as negative regulators of FOXP3 and CTLA4 expression^21^.

In order to identify secreted proteins that regulate FOXP3 and CTLA4 expression, we applied the human Treg flow cytometry phenotypic assay as described^21^. We screened a library of 575 secreted human proteins that has been generated using a high-throughput approach in mammalian cell-factories^22–24^. Four proteins including growth and differentiation factor 7 GDF-7, IL-10, prostatic acid phosphatase PAP and IFNα-7 were identified as positive regulators that increased FOXP3 and/or CTLA4 protein expression. Ten interferon proteins, were identified as negative regulators that reduced the expression of both CTLA4 and FOXP3 proteins without affecting cell viability.

## Results

### Secretome-based screening

In a secretome-based screen a secretome library, consisting of recombinant produced human proteins, is combined with a disease-relevant cell and readout to identify novel biology and signaling pathways (reviewed in^25^). The secretome library is generally produced using high-throughput methods. Proteins that are not feasible to produce will not be included in the library^24^. Conditioned medium^26^ or purified proteins at varying concentrations^22,27^ are typically used as screening sample.

### Production and stratification of the secretome library for the Treg screen

The secretome library was stratified and produced as described in the method section and as described preciously^22^. A mammalian expression system was used for expression and a C-terminal affinity tag was used for purification. Additional, specific stratification was made for the Treg screen^28–31^ creating a specific protein list with a total of 575 proteins included in the screen.

### Identification of secretome protein actives in the human Treg assay by primary screening

**Figure 1:**
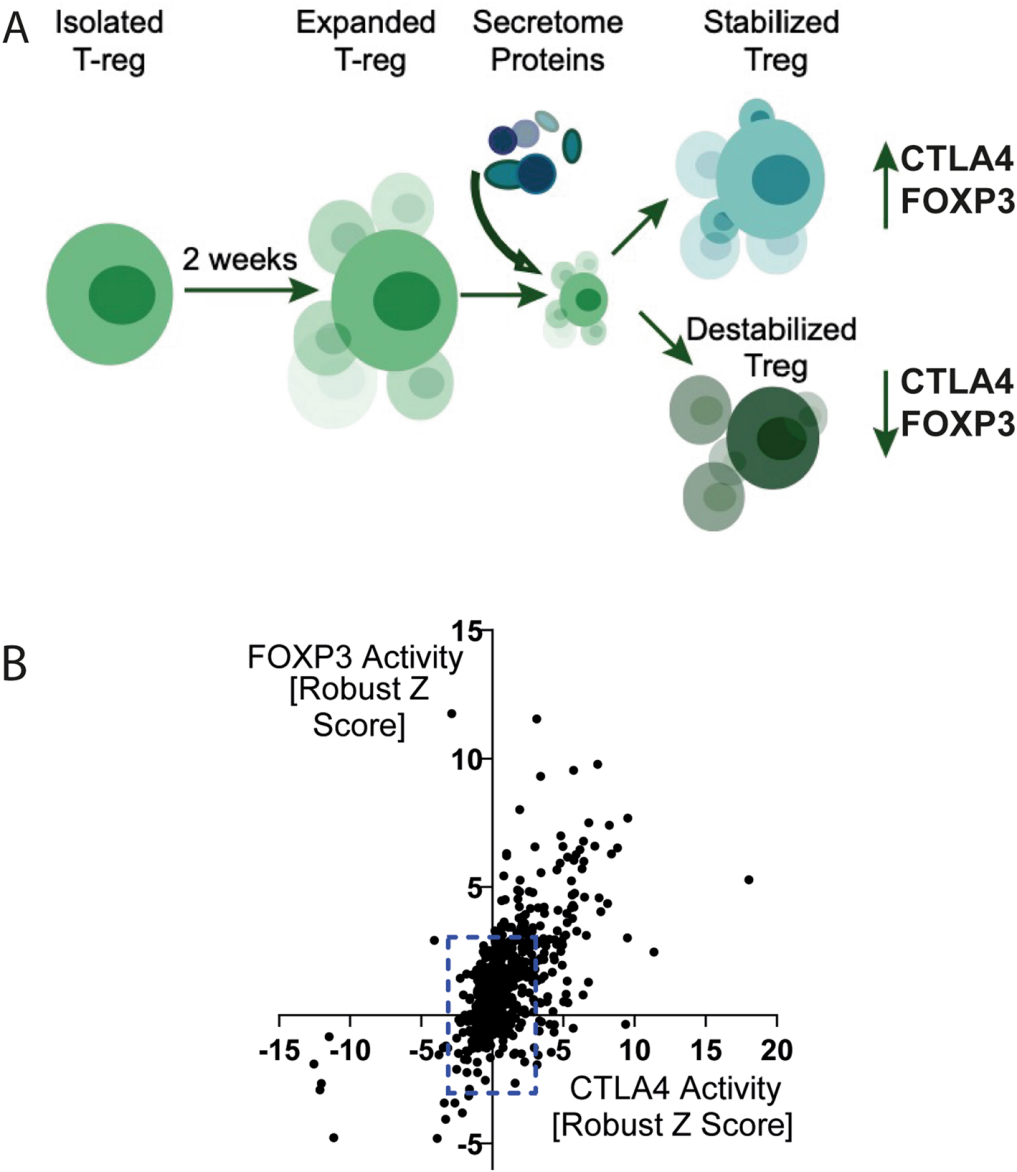
Identification of secreted proteins that stabilise/destabilise Treg markers. (**A**) Schematic describing the concept of the Treg assay where FOXP3 and CTLA4 protein expression was used as an indicator of Treg stability. (**B**) Effect of proteins screened on FOXP3 and CTLA4 expression in Tregs at top concentration tested. The effect was measured as FOXP3 and CTLA4 Median Fluorescence Intensity (MFI) of live cells and normalized to Robust Z Score based on the on-plate PBS neutral controls. The blue dashed lines indicate the threshold for identifying hits based on ≥ 3 × Robust Z-score, or ≤ -3 × Robust Z-score of FOXP3 or CTLA4 expression.

The Treg flow cytometry assay allowed for measurement of multiple parameters, including cell viability, FOXP3 and CTLA4 expression, % FOXP3^+^ cells, % CTLA4^+^ cells, simultaneously. We use the gene names FOXP3 and CTLA4 when we refer to gene expression and use “FOXP3/CTLA4 protein expression” when we specifically refer to expression of the corresponding protein product. 575 proteins (Table S1) were screened in the Treg assay at two concentrations. For most of the proteins, the high concentration was 250 nM and the low concentration was with five-fold dilution, with PBS included as a neutral control. The effect of human secretome proteins on Treg stability was measured as effect on FOXP3 and CTLA4 protein expression (illustrated in Fig. 1A). In the primary screen, secretome protein hits were identified based on ≥ 3 × robust Z-score, or ≤ − 3 × robust Z-score of FOXP3 or CTLA4 expression without negatively affecting cell viability (≥ 85% cell viability required). The method for calculating robust Z-score is described in the methods section under “Data and statistical analysis”. In this screen we identified proteins that increased both FOXP3 and CTLA4 expression, suggesting an improved Treg stability and proteins that reduced FOXP3 and CTLA4 expression, suggesting a destabilised Treg phenotype (Fig. 1B). Secretome protein hits identified from the primary screen were further tested in ten-point concentration response curve experiments using Treg cells from two to three more donors. This resulted in 14 confirmed active proteins showing concentration dependent effects in Treg cells from multiple donors. Four proteins (GDF-7, IL-10, PAP and IFNα-7) increased FOXP3 and/or CTLA4 expression, and 10 proteins (IFNα-4, IFNα-5, IFNα-6, IFNα-8, IFNα-10, IFNα-14, IFNα-16, IFNα-17, IFNα-21, IFNw1) reduced both CTLA4 and FOXP3 without affecting cell viability. The effects induced by GDF-7, IL-10, PAP, IFNα-4, IFNα-7 IFNα-10, and IFNα-14 are discussed in more detail in the following sections. The results of the other 7 proteins IFNα-5, IFNα-6, IFNα-8, IFNα-16, IFNα-17, IFNα-21 and IFNw1 are shown in the supplementary (Figure S1).

### GDF-7 and IL-10 increased FOXP3 and CTLA4 expression and PAP increased FOXP3 expression

**Figure 2:**
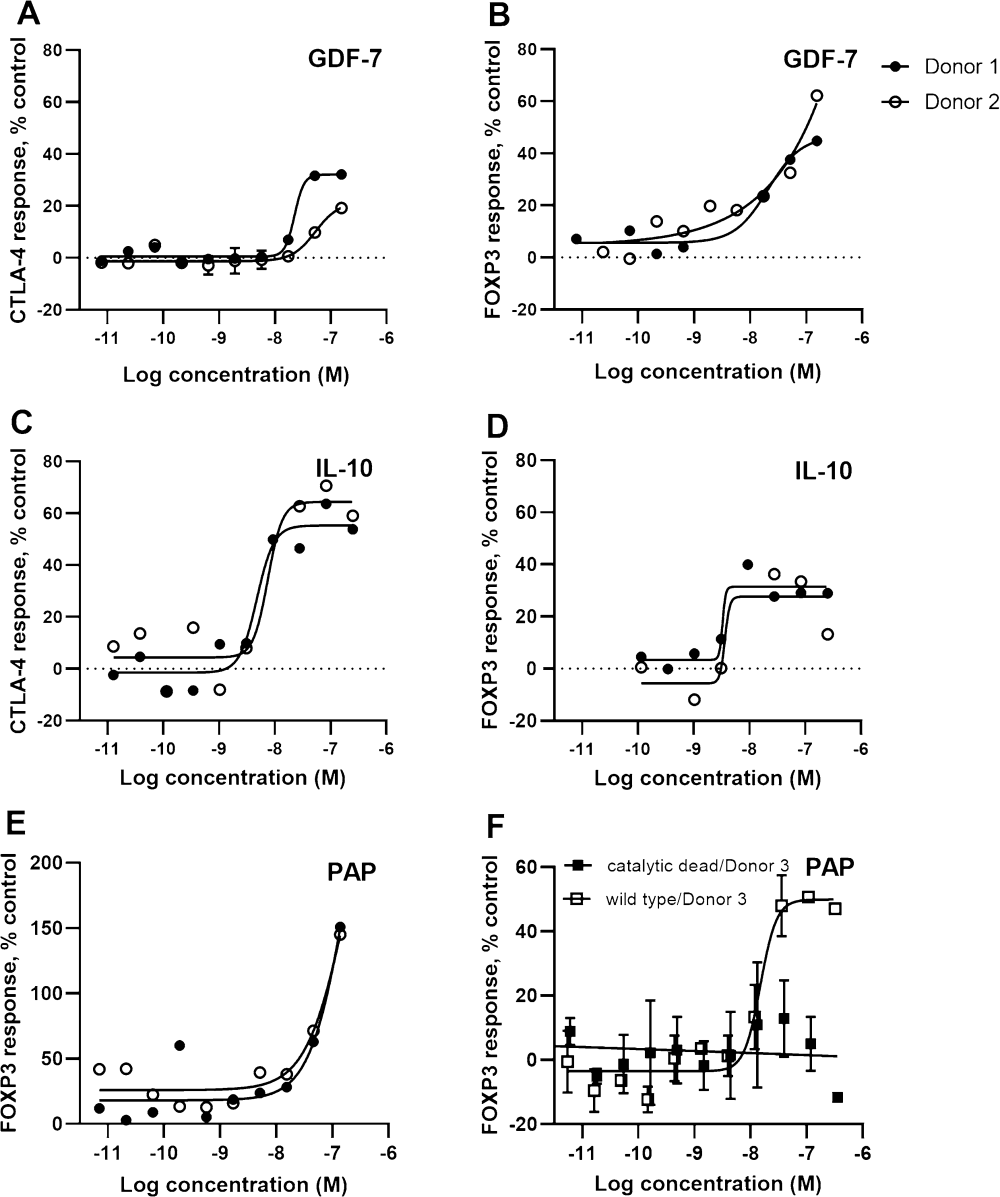
GDF-7, IL-10 and PAP stabilise the Treg markers. The concentration dependent effects on FOXP3 and CTLA4 protein expression induced by GDF-7, IL-10 and PAP are shown in (**A**,**B**) for GDF-7; (**C**,**D**) for IL-10; and (**E**,**F**) for PAP. The effect on FOXP3 and CTLA4 expression was normalized to % activity as compared to the MFI of live cells treated with the on-plate PBS neutral controls. These proteins were tested in human Treg cells from multiple donors (Donor 1 filled circle ●, Donor 2 empty circle ○, Donor 3 empty square □ for wild type PAP, Donor 3 filled square ■ for catalytic dead mutant PAP [H12A]).

Figure 2 shows the increased CTLA4 and FOXP3 expression induced by GDF-7 (Fig. 2A,B), and IL-10 (Fig. 2C,D). Prostatic acid phosphatase (PAP) stabilised the FoxP3 marker (Fig. 2E). Since PAP is a phosphatase^32^, we expressed and purified a catalytic-dead mutant [H12A]^33^ to determine if the observed effect in the primary assay was dependent on catalytic activity. The results showed that the catalytically-dead version of the protein did not induce an increase in FOXP3 expression (Fig. 2F; filled square), in contrast to the wild-type protein (Fig. 2F, empty square). This shows that the PAP-induced effect on Tregs is dependent on phosphatase activity.

### Nine IFNα proteins reduced CTLA4 and FOXP3 expression and one IFNα protein increased CTLA4 expression

**Figure 3:**
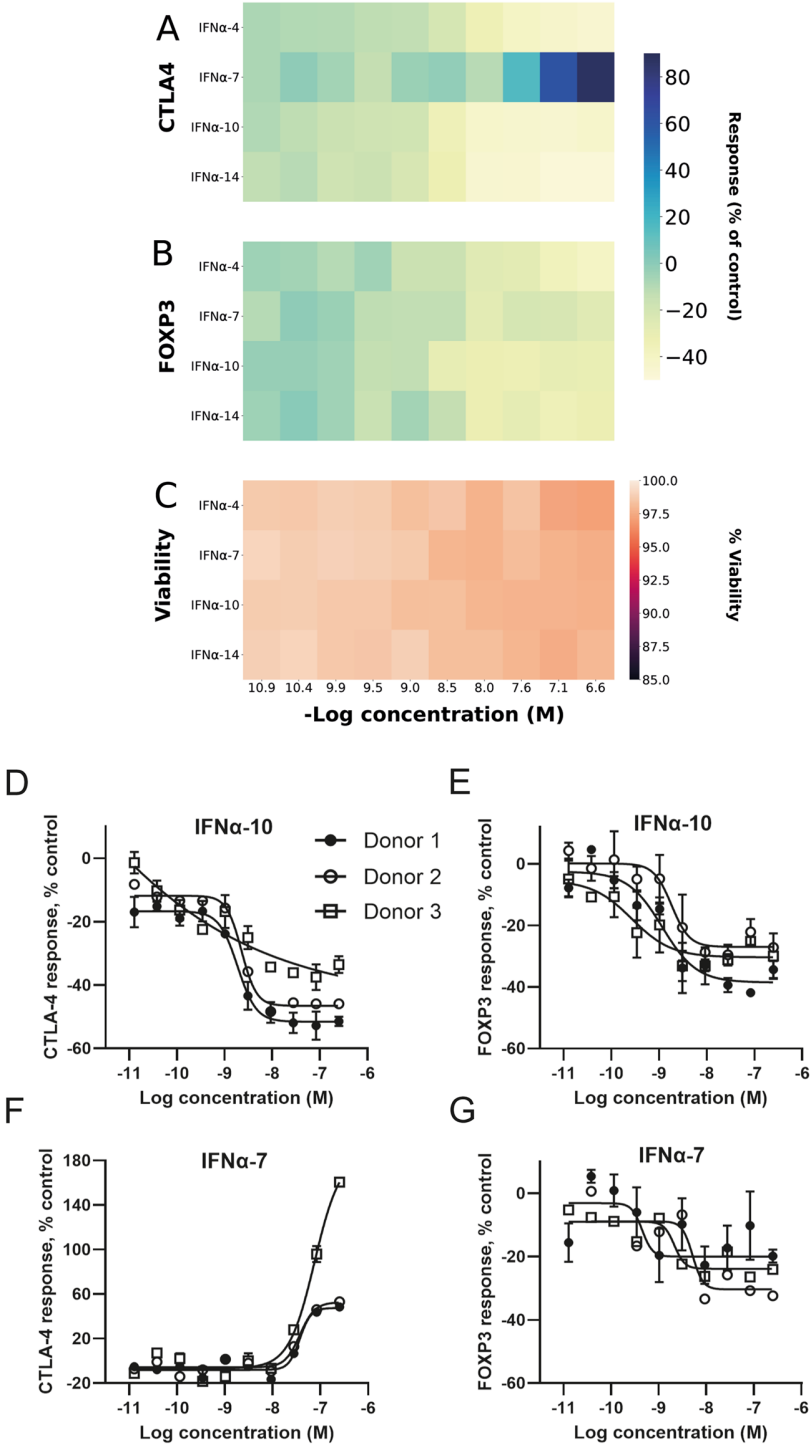
IFNα-4, IFNα-10 and IFNα-14 destabilise Treg markers whereas IFNα-7 stabilises CTLA4 expression. (**A**) Heatmap over mean effect on CTLA4 protein expression, (**B**) heatmap over mean effect on FOXP3 protein expression, (**C**) Heatmap over mean effect on cell viability, (**D**–**E**) Effect of IFNα-10 treatment on CTLA4 and FOXP3 expression, (**F**–**G**) Effect of IFNα-7 treatment on CTLA4 and FOXP3 expression. Tregs were from multiple donors (Donor 1 filled circle ●, Donor 2 empty circle ○, Donor 3 empty square □). The effect on FOXP3 and CTLA4 expression was normalized to % activity as compared to the MFI of live cells treated with the on-plate PBS neutral controls.

Figures 3 and S1 show the effect of the IFNα family of proteins on FOXP3 expression and CTLA4 expression. In total, ten IFNα family-members were identified as regulators of the two Treg markers. Nine proteins reduced expression of both FOXP3 and CTLA4. IFNα-7 increased CTLA4 and reduced FOXP3 expression. Figure 3A,B show heatmaps for IFNα-4, IFNα-7, IFNα-10 and IFNα-14 on FOXP3 expression and CTLA4 expression. Heatmaps for IFNα-5, IFNα-6, IFNα-8, IFNα-16, IFNα-17, IFNα-21 and IFNw1 are shown in Fig. S1. Treg cell viability was not affected by treatment with the IFNα proteins (Fig. 3C and Fig. S1). Concentration responses for IFNα-7 and IFNα-10 effects on FOXP3 and CTLA4 expression are shown in Fig. 3D–G.

### Differential effect on Treg cells gene expression induced by IFNα-7 versus IFNα-10

Interestingly, IFNα-7 showed a different effect on Tregs compared to the other IFNαs. IFNα-7 strongly increased CTLA4 protein expression, whereas the other IFNαs reduced CTLA4 protein expression. To further understand the differential effects, we performed RNAseq analysis on Tregs treated with IFNα-10 and IFNα-7. Table S2 summarises the data from the differential gene expression analysis.

**Figure 4:**
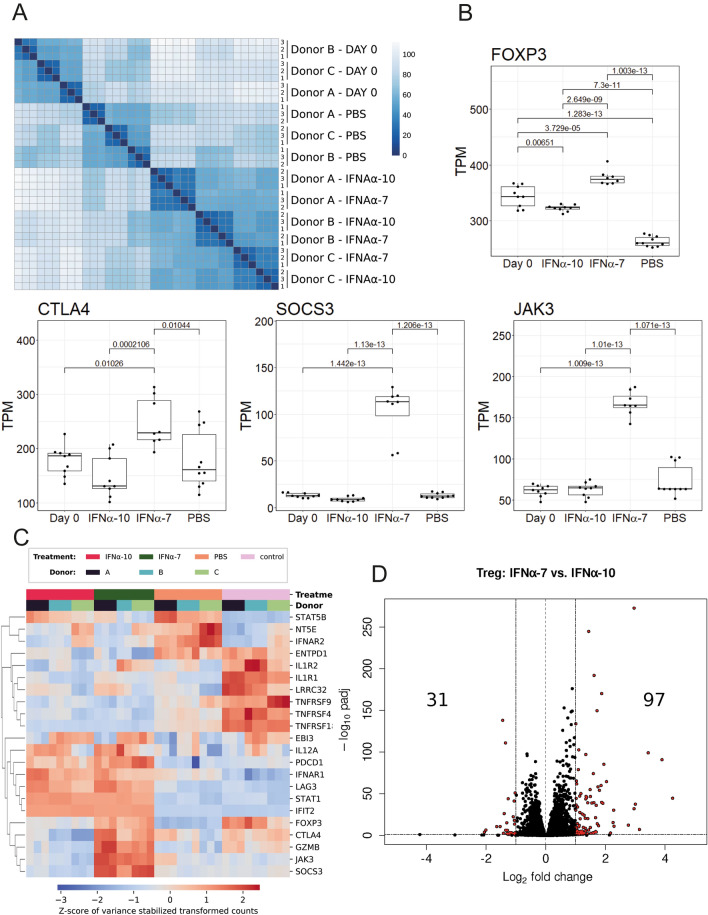
Transcriptomics reveal distinct activation profile for IFNα-7 compared to IFNα-10. (**A**) Heatmap of the sample-to-sample euclidean distances for the RNA samples, based on variance stabilised counts data. (**B**) Expression analysis (TPM) shows that FOXP3, CTLA4, SOCS3 and JAK3 genes were upregulated when comparing IFNα-7 treated to IFNα-10 treated cells. (**C**) Heatmap showing differences in expression between samples for genes mentioned in the results section. Values were based on transformed count data (variance stabilising transformed) extracted from DESeq2 with its vst function and with blind set to FALSE. Z-scores were subsequently applied to accentuate differences between samples. (**D**) Differentially expressed genes (twofold change and FDR < 0.05) when comparing IFNα-7 and IFNα-10 treatments of Treg cells; 97 genes were significantly upregulated and 31 genes were downregulated with this threshold. Heatmap of the sample-to-sample euclidean distances for the RNA samples, based on variance stabilized counts data**.** Treg cells from 3 individual donors were analysed in the transcriptomics experiments in 3 replicates from each individual donor.

A heatmap of the sample-to-sample distances showed a high correlation between the two different IFNα treatments of Tregs (Fig. 4A). FOXP3 gene expression showed a significant reduction in Tregs treated with PBS during the 24–26 h treatment compared to Tregs from the day 0 control (Fig. 4B). In the day 0 control, cells were collected on the plating day without receiving any further treatment. This indicated that Treg cells were unstable over time in vitro*.* Other Treg marker genes that showed a significant and considerable fold change (padj < 0.05, twofold change) in expression between the day 0 control and the PBS samples (Table S2), and are mentioned in this section, are displayed in the heatmap in Fig. 4C. Genes include, NT5E (upregulated in the PBS samples) and TNFRSF18, ILR1, GZMB, LRRC32, EBI3, IL1R2, TNFRSF4, and TNFRSF9 (downregulated in the PBS samples). Both IFNα-7 and IFNα-10 significantly increased FOXP3 gene expression compared to PBS (Fig. 4B). Treatment with IFNα-7 significantly increased CTLA4 gene expression (Fig. 4B). IFNα-10 slightly reduced CTLA4 gene expression (although not significantly) compared to PBS control treatment (Fig. 4B). The two IFNα treatments significantly increased expression of the known Treg marker genes EBI3, IL12A, PDCD1, and LAG3 while the expression decreased for TNFRSF18 (padj < 0.05, twofold change, Fig. 4C). Treg associated genes NT5E, TNFRSF9, and ENTPD1 were downregulated when comparing IFNα-7 to PBS treatment but not for IFNα-10 versus PBS, using the same cut-off (Fig. 4C). Nevertheless, these genes were also significantly downregulated for IFNα-10. In total, the two IFNα treatments showed 128 differentially expressed genes (padj < 0.05, twofold change) when compared to each other (Fig. 4D, Table S2).

Additionally, SOCS3 (an inhibitor of STAT signaling) and JAK3 were among the differentially expressed genes, with SOCS3 as the third most over-expressed gene in IFNα-7 compared to IFNα-10, showing more than ten times higher expression in IFNα-7 (Fig. 4B). Likewise, a gene set analysis showed a significant change in IL2 STAT5 signaling when comparing cells treated with either IFNα-7 or IFNα-10 (Fig. S2). This analysis also showed significant differences in inflammatory response, IL6 JAK STAT signaling, interferon gamma and interferon alpha response. Furthermore, IFNα-7 treated cells displayed a clear upregulation in unfolded protein response, which indicated an elevated cellular stress response associated with the endoplasmic reticulum in cells treated with IFNα-7 compared to IFNα-10. A further analysis of the transcriptome in the Tregs, specifically focusing on the STAT family of proteins with genes annotated in Pathway Studio to have their expression either positively or negatively regulated by STATs was performed. This gene set variation analysis (GSVA) was used to evaluate the involvement of the respective STATs. The analysis showed that genes which are annotated to be negatively regulated by STAT1 were down-regulated by IFNα-10. Genes shown to be positively regulated by STAT5B were upregulated by IFNα-7 (Fig. S3).

**Figure 5:**
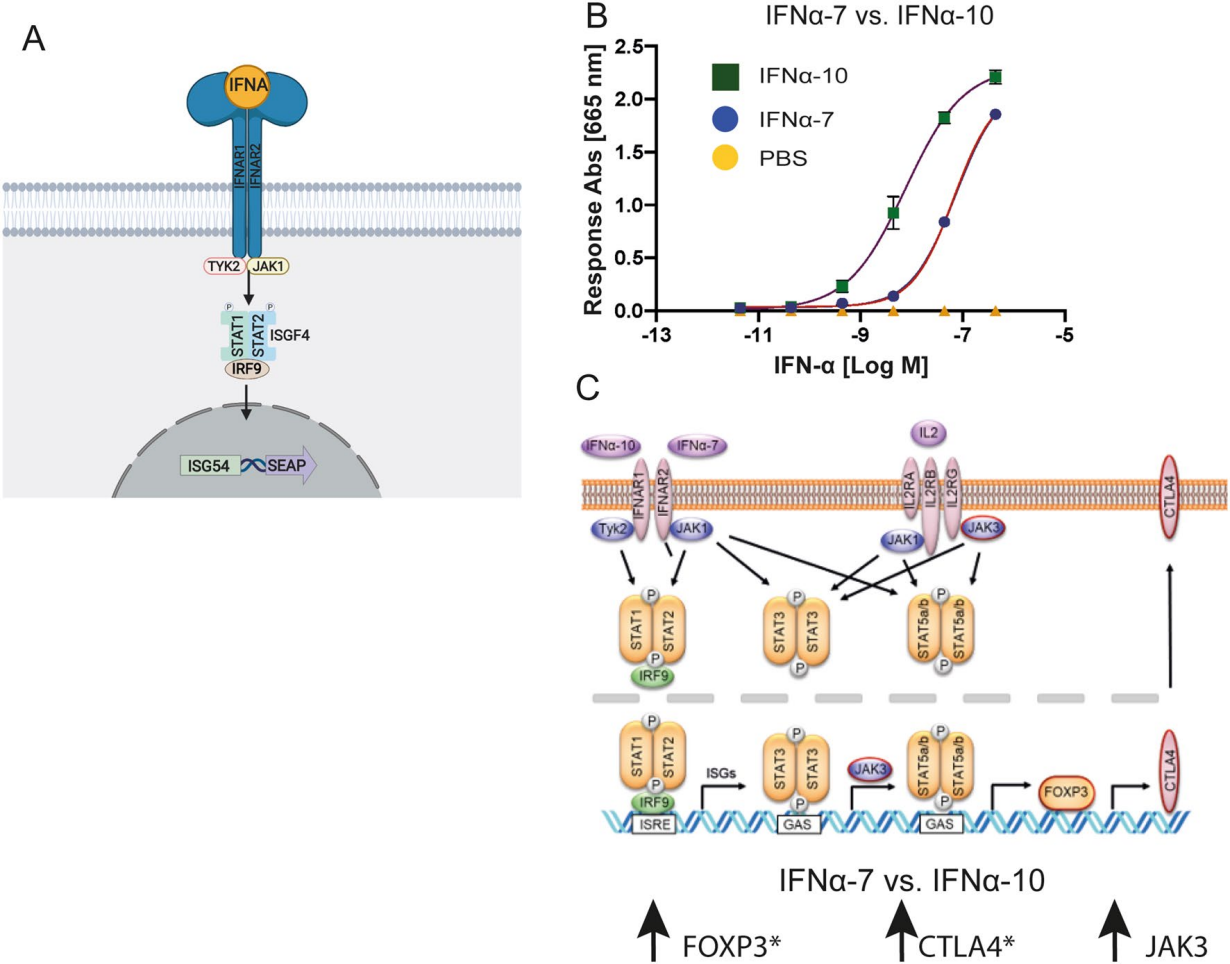
Suggested model of IFNα-7 vs IFNα-10 strengthens difference in ISG transcription signaling. (**A**) Schematic representation of HEK293 reporter cell line for IFNAR1/2 mediated JAK STAT1/2 signaling leading to ISG transcription of secreted embryonic alkaline phosphatase. (**B**) Dose–response curves of HEK293 reporter cell line upon treatment with either IFNα-7 (blue circles) or IFNα-10 (green squares), SEM error bars based on n = 2, duplicate measurements and subtracted baseline signal (PBS). (**C**) A pathway map showing signaling pathways for the IFNAR1/IFNAR2 receptor and the IL-2 receptor. Genes in red are upregulated when comparing IFNα7-treated versus IFNα10-treated cells. JAK3 is significantly upregulated (FDR < 0.05 and > twofold upregulation). * indicates additional phenotypic readouts at a protein level in the primary assay. The observed differences between IFNα-7 and IFNα-10 are further discussed in the main text.

A simplified model HEK293 based reporter system (Fig. 5A) was used to further investigate the potency difference in JAK / STAT signaling between IFNα-7 and IFNα-10. Activation of signaling, in this model system, results in IFNAR1/2 activated downstream ISG54 transcription of secreted embryonic alkaline phosphatase. The reporter cells were treated with different concentrations of IFNα-7 and IFNα-10 in a dose response experiment. Treatment resulted in a ten-fold weaker signaling for IFNα-7 (3.3 nM) over IFNα-10 (0.3 nM), as monitored by EC50.

### Expression analysis of receptor genes on Tregs

The RNAseq data set enabled us to interrogate expression of receptors that could be relevant for the data interpretation. The RNAseq analysis showed some expression of BMPR1A and BMPR2 but a very low expression of BMPR1B on the Tregs at day 0 and day 1 (Fig. S4). The RNAseq analysis showed that ADORA2A/A2a gene expression was equally abundant at day 0 and day 1 (Fig. S4). Expression of IFNAR2 significantly increased, comparing day 0 and day 1 PBS-control, while IFNAR1 did not. Both treatment with IFNα-7 and IFNα-10 proteins prevented this increase (Fig. S5).

## Discussion

Treg cells have been characterised as the key cell type controlling peripheral autoreactive T lymphocytes. Treg cell function and FOXP3 expression are recognised to be important for autoimmune and inflammation diseases. However, molecular targets regulating FOXP3 expression and stability of Treg cell function are not well established^2,3,34,35^. Previous studies have demonstrated the importance of secreted proteins for regulating Treg cell function^16,17,36^. Here, we have successfully screened 575 secreted proteins in a previously established Treg flow cytometry assay and identified secreted proteins that regulate FOXP3 and CTLA4 expression. Our screening identified: 1) four secretome proteins, including GDF-7, PAP, IFNα-7 and IL-10 (a well-known cytokine required for maintaining the Treg phenotype), that increased FOXP3 and/or CTLA4 expression and 2) ten interferon proteins, that reduced the expression of both FOXP3 and CTLA4. This simplified system can be used to assess Treg stability. An increase in Treg FOXP3 and CTLA4 expression would stabilise the Treg cell phenotype and benefit patients with autoimmune disease. Aa decrease in Treg cell FOXP3 expression would destabilise the Treg cell phenotype and attenuate their suppressive activity and enhance immune responses to tumours in patients with cancer^37^.

Growth and differentiation factor 7 (GDF-7) belongs to the TGFβ superfamily of proteins. The importance of signaling via the TGFβ-pathway for the maintenance of peripheral Treg cells is well documented^38^. GDF/BMP proteins bind to a heteromeric receptor consisting of a type 1 and type 2 heterodimer. There are some reports describing receptor preferences for GDF proteins including GDF-7^39^. Possible receptors could be a combination of the type 1 receptor BMPR1A or BMPR1B with BMPR2. The RNAseq analysis showed some expression of BMPR1A and BMPR2 but a very low expression of BMPR1B on the Tregs at day 0 and day 1 (Fig. S4). These results indicate BMPR1A and BMPR2 as potential receptors for GDF-7 in Treg cells.

Acidic prostate phosphatase (PAP) is highly expressed in serum from prostate cancer patients and has been used as a diagnostic marker (reviewed by^40^). The enzyme is an acidic phosphatase with unspecific substrate specificity. The purified enzyme also displays nucleotidase activity in vitro and converts AMP into free adenosine^32^. Despite its name PAP is also expressed in other tissues including thymus, lymph nodes and spleen^32,41^. The data presented here, showed that PAP stabilised the Treg FOXP3 marker and that stabilisation is dependent on the enzymatic activity of the PAP-protein. Adenosine is a well-known effector of immuno-suppression^42^. A well-known mechanism for generation of free adenosine is by hydrolysis of ATP/ADP and AMP by CD39 and CD73, respectively. Both CD39/ENPTD1 and CD73 are surface markers for murine Tregs. On human Tregs, it has been reported that the expression of CD39 shows a high inter individual variation and is upregulated at sites of inflammation^43^ and the co-expression of CD73 on CD39+ Tregs is very rare^44^.The generated adenosine signals via the ADORA2A/A2a receptor^42^. Although CD73 is the major enzyme for conversion of AMP to adenosine, emerging data is suggesting that PAP could also play a role (reviewed by^45^). A double knockout of CD73 and PAP displayed reduced percentages of Tregs in the lymph nodes and thymus^32^. Such a role for PAP strengthens our hypothesis that the stabilising effect on Tregs is due to generation of adenosine by PAP leading to a signaling effect via the A2a receptor that stabilises the Treg phenotype. The RNAseq analysis shows that the ADORA2A expression is equally abundant at day 0 and day 1 (Fig. S4).

Type I interferons are suggested to play essential roles in both host defence and as critical mediators of autoimmune disease (^46,47^). However, their effect on Tregs is contradictory since both positive effects on Treg expansion and/or function as well as inhibitory roles in Treg proliferation and suppressive function have been reported^19,48,49^. The conflicting results could be partially explained by the complex networks between the Treg intrinsic and Treg extrinsic effects. In addition, increased evidence has shown that human Tregs are not homogenous and different subsets of Tregs can have different functions^13^. Our results showed that treatment with a variety of IFNαs, including IFNα-4, IFNα-10 and IFNα-14, significantly reduced the protein expression of FOXP3 and CTLA4. These treatments also reduced the numbers of FOXP3^+^ and CTLA4^+^ cells relative to PBS neutral control treated Treg cells. Campell et al*.* demonstrated that type I interferons inhibit co-stimulation-dependent Treg cell activation and proliferation, both in vitro and in vivo during acute infection with lymphocytic choriomeningitis virus^50^. It has been reported by Ganaplara and colleagues that a lack of IFNα receptor signaling induced higher number of activated Treg during viral infection^12^. Our results are consistent with Campell and Ganaplara et al*.*, and indicate that the IFNα family negatively regulates Treg cells.

Granzyme B has been described to be important for the Treg suppressor function during virus infection^51,52^ and specifically in a RSV infection model^51^. Granzyme B was eightfold upregulated in IFNα-7 treated Tregs compared to IFNα-10 treated cells and PBS-treated controls. Other proteins associated with Treg suppressor function such as CTLA4 and ICOS were also upregulated (Table S2). To our knowledge, this is the first time it has been shown that IFN-α7 in contrast to other type I interferons increase markers associated with Treg suppressor function.

Interestingly, treatment with IFNα-7, which binds to the same hetero-dimeric IFNAR1 and IFNAR2 receptor as the other IFNα proteins^53^, resulted in a different effect on Treg as shown by increased CTLA4 protein expression. Moreover, the differential expression analysis showed that JAK3 and SOCS3 were significantly increased in IFNα-7 treated Tregs, compared to IFNα-10 treated Tregs. Genes which are annotated to be negatively regulated by STAT1 were more down-regulated by IFNα-10 whereas genes shown to be positively regulated by STAT5B were upregulated by IFNα-7. These results demonstrate the differential effect on Treg cells induced by IFNα-7 versus IFNα-10 treatment. The interactions of IFNαs with the IFNAR mediates activation of JAKs to induce the phosphorylation of STAT1/STAT2. While the difference in phenotype could be due to a direct on/off switching of pathways, our HEK293-model reporter experiment focusing on the IFNAR1/2 JAK/STAT pathway suggests it rather to be a significant nuance change in JAK/STAT signaling (tenfold) than complete on off switching of this pathway. All in all, the observations made from the differences in signaling between IFNα-7 and IFNα-10 appear to have shifted the balance in downstream JAK/STAT activation with elevated gene expressions of FOXP3, CTLA4, SOCS3 and JAK3 levels (Fig. 5C). Whether these nuances in expression levels affect phosphorylation of other neighbouring pathways remains to be investigated. In addition, IFNAR signaling can trigger phosphorylation of other STATs, such as STAT3 and STAT5A/B^54^. STAT5A/B are transcription factors inducing FOXP3 expression and FOXP3 is a transcription factor inducing expression of CTLA4^55^ which is in line with the suggested overall model of the gene expression difference (Fig. 5C). The observed difference could also be by enhancing IL-2 receptor signaling. IL-2 is present in the cell media during the Treg screen. JAK3, which was upregulated in IFNα-7 treated Treg cells, is an essential component in IL2 receptor signaling^56^.

The group of Schreiber et al.^53^ have studied the interactions of type 1 interferons with IFNAR1 and IFNAR2 receptors, by using biochemical and structural studies. They have demonstrated that the interactions of type 1 interferons with IFNAR1 and IFNAR2 are weak and tight, respectively, based on IFNα-2 and IFNw1 ligand-receptor complexes. However, there is no detailed structural or biochemical information available for IFNα-7 and IFNα-10. In addition, the different IFNα members are suggested to have differential affinities for the two receptors^53^. Additional analysis using Tukey’s range test shows that expression of IFNAR2 significantly increases, comparing day 0 and day 1 PBS-control, while IFNAR1 does not. Both IFNα-7 and IFNα-10 prevented this increase. Thus, we cannot explain the difference is signaling by a differential effect on IFNAR gene expression (Fig. S5). However, the differential effect could be explained by that IFNα-10 has a higher affinity for the IFNAR2 molecule as suggested by the expression profile at day 0. This would need to be tested experimentally.

In the current study, we use Treg cells from two donors in the secretome primary screen. We continue by hit confirmation in Treg cells from two to three additional donors. The RNAseq study is performed in Tregs from three additional donors. A useful next step could be to further study the effect of the confirmed secretome hits in Tregs from additional biological donors and from selected donors grouped for example by age and/or gender.

In summary, this is the first report of screening a secretome library for the identification of secreted proteins that regulate FOXP3 and CTLA4 expression in human Treg cells. Screening a secretome library in the Treg cell phenotypic screen allowed us to identify protein signals that regulate Treg cell marker proteins FOXP3 and CTLA4 expression and Treg cell phenotype stability. The identified proteins are valuable tools for understanding the biology of Treg cells and have potential implications for establishing novel therapies for autoimmune diseases and cancer.

## Methods

### Ethics statement

The study was conducted ethically, with consent for research, in accordance with all regulatory requirements from Swedish Ethics committee and the AstraZeneca Biobank in Sweden. The collection and use of human blood samples from healthy volunteer donors at AstraZeneca was performed with approval from Swedish Ethics committee in Gothenburg (reference number Ad 033-10). Participants were informed about the study and written informed consent was obtained from participants. All procedures followed appropriate guidelines.

### Stratification, production of proteins and sample management

General stratification of the secretome library was performed as detailed previously^22^. Additional, specific stratification was made for the Treg screen^28–31^ creating a specific protein list with a total of 575 proteins included in the screen. Secretome proteins were expressed in Chinese Hamster Ovary (CHO) EBNALT 85 cells using the QMCF Technology (Icosagen Cell Factory OÜ, Tartu, Estonia). A C-terminal HPC4 tag was used for affinity purification using an anti-HPC4 coupled resin and mild elution with EDTA^22^. Proteins were desalted. Average concentration of the produced proteins after desalting was 13 µM. Proteins that had a concentration above 13 µM were diluted. The proteins were aliquoted before they were snap-frozen in liquid nitrogen and stored at − 80 °C. Stock concentrations of proteins included in the tested library were at measured concentrations that varied between 2–13 µM (Table S1). Quality control included protein identification by MS/MS, purity by SDS-PAGE. Endotoxin levels were generally < 0.5 EU/mL^22^. Human PAP and a catalytic-dead mutant form of PAP [H12A] were expressed using an Expi293™ expression system (ThermoFisher, Cat#A1452) using the pEBNAZ vector^57^ and a C-terminal His-tag for purification with Ni–NTA chromatography followed by size exclusion chromatography.

### Peripheral blood mononuclear cell (PBMC) isolation

Human blood samples were collected in heparin tubes as anonymized samples from consenting healthy volunteer donors at AstraZeneca (with approval for collection of the samples from the Ethics committee in Gothenburg, Sweden, Ad 033-10) and PBMCs were prepared from blood by density centrifugation, using Lymphoprep (Stemcell Technologies) and Sepmate tubes (Stemcell technologies, Cat#15450) according to manufacturer’s protocol.

### Human Treg cell isolation and expansion

Treg cells were isolated from human PBMCs and expanded as described by Ding et al.^21^. Briefly, CD4+ T cells were negatively sorted using CD4+ T Cell isolation kit (130-096-533, Miltenyi Biotech). Enriched CD4+ T cells were stained with conjugated antibodies CD4 PerCP (1:10, 550,631, BD Bioscience San Jose, CA, USA), CD127 BV421 (1:10, 562,436, BD Bioscience), CD25 PE-Cy7 (1:10, 557,741, BD Bioscience) and CD45RO APC (1:10, 559,865, BD Bioscience) before Tregs (CD4+ CD25highCD127lowCD45RO-) were sorted on a BD Aria FACS II (BD Biosciences). The gating strategy for sorting naïve CD4^+^CD25^high^CD127^low^CD45RO^−^ Treg cells is shown in the supplementary (Fig. S6A). For all donors, the purity of FACS sorted naïve CD4^+^CD25^high^CD127^low^CD45RO^−^ Treg cells was above 90%. Sorted Tregs were expanded for 7 days at 37 °C using Treg expander beads (11129D, ThermoFisher) at a bead to cell ratio of 4:1 in expansion culture media (RPMI 1640 Glutamax-1, ThermoFisher + 10% heat inactivated FCS + 100 U/mL PEST + 1 mM sodium pyruvate) supplemented with 10 ng/mL recombinant human IL-2 (rhIL-2) (200-02, Peprotech, Rocky Hill, NJ) and 100 nM Rapamycin (R8781, Sigma Aldrich). After 7 days of expansion, beads were magnetically removed and Rapamycin was washed away. Tregs were expanded for another 7 days in 10 ng/mL rhIL-2 containing culture media.

### Human Treg flow cytometry phenotypic assay

The Treg flow cytometry assay was performed as described by Ding et al.^21^. Briefly, expanded Treg cells were collected and counted on a System XT-1800i (System Corporation, Kobe, Japan), pelleted by centrifugation at 300 g, resuspended and seeded at a density of 4000 cells/well in 50 µL of rhIL-2 containing culture media in 384-well round-bottom ultra-low attachment plates (Corning Inc Cat#3830). 1 µL of secretome proteins (concentrated stock varying between 2–13 µM and a majority of proteins at 9–13 µM or PBS diluted) or neutral control PBS were added to individual wells. This corresponded to proteins being tested in intervals of 50–250 nM and 10–50 nM for high and low concentrations, respectively. Assay plates containing Tregs and secretome proteins were incubated at 37 °C and 5% CO2 for 72 h. Assay plates were then stained with Live/Dead fixable Aqua dead cell stain kit (L34966, ThermoFisher), fixed and permeabilized with FOXP3/Transcription Factor Staining Buffer Set (00–5523, eBioscience ThermoFisher), then stained for FOXP3 and CTLA4 with conjugated antibodies FOXP3 PE (1:20, 560,046, BD Bioscience) and CD152 APC (1:20, 555,855, BD Bioscience) using a Beckman Coulter automation system as described previously^21^. Stained cells were washed, resuspended in FACS buffer (PBS + 2% FCS), and sampled on an automated high throughput flow cytometry platform iQue Screener PLUS (IntelliCyt) using a reading protocol with a 10 s sip time per well, 4 s shake at 2800 rpm after every 6 wells and a mid-plate clean after every 48 wells. The iQue flow cytometry data was processed and analysed using ForeCyt software v6.0 (IntelliCyt) for well identification, compensation, gating of populations and generation of parameters of interest. The raw data from the Treg flow cytometry phenotypic screen was collected as cell counts, Median Fluorescence Intensity (MFI) at VL2-H (Ex 405 nm, Em 530/30 nm) for identifying live and dead cells, MFI at BL2-H (Ex 488 nm, Em 572/28 nm) for FOXP3 expression and MFI at RL1-H (Ex 640 nm, Em 675/30 nm) for CTLA4 expression. The gating strategy for FOXP3^+^, CTLA4^+^ live cells is shown in the supplementary (Fig. S6B). For all donors, the percentage of FOXP3^+^ live cells and CTLA4^+^ live cells of PBS neutral control treated cells was above 70%. The cell viability of PBS treated cells was above 97% after expanded cells were treated with PBS for 3 days (Fig. S7).

### Treg RNAseq experiments and analysis

Expanded Treg cells from 3 individual donors were seeded at a density of 800 000 cells/well in 1000 µL of rhIL-2 containing culture media in 24-well plates (3524, Corning) and treated with IFNα-7, IFNα-10 or PBS neutral control at 37 °C and 5% CO2. After a 24–26 h incubation, cells were washed with PBS and lysed with RNeasy cell lysis buffer RLT (RNeasy Mini kit, 74,104, Qiagen, Copenhagen, Denmark). For day-0 control, 1000 000 cells per sample from each donor were collected at the cell plating day without any treatment. Total RNA from treated cells and day-0 control was purified with RNeasy Mini kit using a RNA Cell Method with DNase digest protocol and 30 µL elution volume on a robotic RNA purification system QIAcube (Qiagen) following the instructions from the manufacturer. Purified RNA samples were stored at − 80 °C until analysis. All RNA samples were analysed on an Agilent 2100 Bioanalyzer prior to sequencing using an Agilent RNA 6000 Nano kit (Agilent Technologies, Santa Clara, CA, US). Only the samples with RIN values > 8 were sequenced. RNAseq of the Treg samples was done by sequencing strand-specific cDNA libraries using a read length of 2 × 150 bp on Genome Sequencer Illumina HiSeq. Sequencing was carried out at GATC Biotech Ltd (Konstanz, Germany). Kallisto^58^ with cDNA human reference (GRCh38, Ensemble release 92) as FASTA file was used to quantify TPMs and counts (included in Supplementary Table 2). The R package DESeq2^59^ with transcript counts was used for differential expression analysis. PIANO^60^ in combination with the MSigDB hallmark gene sets^61^ with the adjusted p values and Log2 fold changes from the differential expression analysis was used for the gene set analysis in Fig. S2. Clustering of correlation matrices was based on rlog transformed counts and the hierarchal clustering was calculated using Euclidean distances. Gene set variation analysis (GSVA) on Treg RNAseq data was performed on TPM expression values using the GSVA R package^62^. Pathway maps were built using the Pathways Studio software. Pathway Studio was also used to extract genes annotated to be directly regulated by Signal transducer and activator of transcription (STAT) transcription factors. Tukey's range test on gene expression values and GSVA enrichment scores were performed in RStudio version 1.1.383 using the TukeyHSD R package.

### IFNα signaling in an in vitro cell reporter assay

The IFNα responsive cell line HEK-BLUE IFN-αβ was purchased from InvivoGen (Toulouse, France) and cultivated in the recommended cultivation media by the manufacturer. All IFNα stimuli assays were performed in the described test media from the cell line according to manufacturer’s instruction. Briefly dilution series of recombinant IFNα proteins were incubated with the HEK-BLUE IFNα responsive cell line seeded to the wells of a 96 well plate at a density of 280,000 cells/mL in 180 µL test medium and plates were incubated for 24 h at 37 °C in a humidified atmosphere with 5% CO2. Supernatant from the cell cultures were incubated with pre-warmed BLUE-substrate for 1.5 h at 37 °C in a humidified atmosphere with 5% CO2 according to manufacturer’s instruction InvivoGen (Toulouse, France) for the quantification of secreted alkaline phosphatases in response to IFNα stimulation. Colour metric read out was performed by absorbance measurements at OD 655 nm utilizing Clariostar plate reader (BMG LABTECH, Offenburg, Germany).

### Data and statistical analysis

Replicates and number of donors are described in the results and figure legends. Results are expressed as means values ± standard error from 2–3 replicate samples unless otherwise indicated in figure legends. Compound concentration response data was analysed in GraphPad Prism 7 using non-linear regression and fitted to a sigmoidal four parameter equation. Box plots of gene expression and GSVA enrichment scores were plotted using the R package ggplot2. Lower and upper hinges correspond to the first and third quartiles. The upper whisker extends from the hinge to the largest value no further than 1.5 * IQR from the hinge (where IQR is the inter-quartile range, or distance between the first and third quartiles). The lower whisker extends from the hinge to the smallest value at most * IQR of the hinge. The centre line marks the median value. Robust Z-score normalization obtained by median and median absolute deviation (MAD) methods is calculated as (X_compound_-X_median_)/MAD_,_ where X_compound_ is the FOXP3 MFI or CTLA4 MFI of the individual compound treatment, X_median_ is the median value of the FOXP3 MFI or CTLA4 MFI of cells treated with the PBS controls, MAD is the median absolute deviation of the FOXP3 MFI or CTLA4 MFI from the PBS treated cells on each 384-well plate^63^.

## Supplementary Information

Supplementary Figures.

Supplementary Table 1.

Supplementary Table 2.
