# Supplementary material for: Secretome screening reveals immunomodulating functions of IFNα-7, PAP and GDF-7 on regulatory T-cells
Source: Sci Rep. 2021 Aug 18;11:16767. doi: 10.1038/s41598-021-96184-z (PMC8373891; doi:10.1038/s41598-021-96184-z)
Supplement: Supplementary file 1 — Supplementary Figures. [file 41598_2021_96184_MOESM1_ESM.docx]

Supplementary material

**Secretome screening reveals immunomodulating functions of IFNα-7, PAP and GDF-7 on regulatory T-cells**

Mei Ding^a,1^, Rajneesh Malhotra^b^, Tomas Ottosson^b^, Magnus Lundqvist^c^, Aman Mebrahtu^c^, Johan Brengdahl^a^, Ulf Gehrmann^b^, Elisabeth Bäck^d^, Douglas Ross-Thriepland^e^, Ida Isaksson^f^, Björn Magnusson^a^, Kris F. Sachsenmeier^g^, Hanna Tegel^c^, Sophia Hober^c^, Mathias Uhlén^c^, Lorenz M. Mayr^e^, Rick Davies^e^, Johan Rockberg^c^ and Lovisa Holmberg Schiavone^a,1^

**Fig. S1.** **Seven interferon proteins destabilize Tregs.** These 7 interferon proteins are IFNα-5, IFNα-6, IFNα-8, IFNα-16, IFNα-17, IFNα-21, IFNw1. (A) Heatmap over mean effect on CTLA4 protein expression in Tregs from multiple donors, (B) heatmap over mean effect on FOXP3 protein expression in Tregs from 2-3 donors, (C) Heatmap over mean effect on cell viability in Tregs from multiple donors. The effect on FOXP3 and CTLA4 expression was normalized to % activity as compared to the MFI of live cells treated with the on-plate PBS neutral controls.


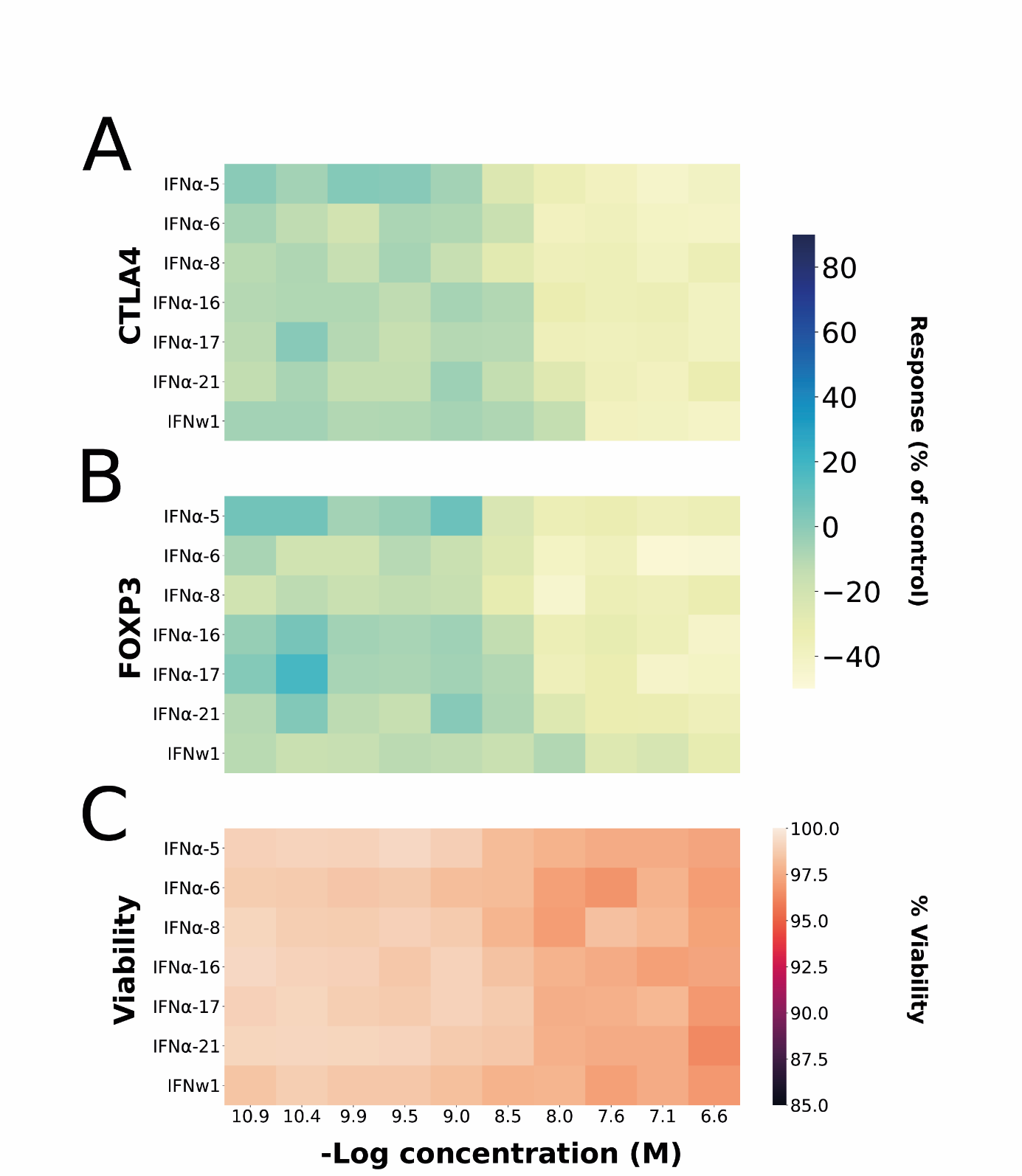


**Fig. S2. Gene set analysis** Gene set analysis using PIANO together with the MSigDB hallmark gene sets and the adjusted p values and Log2 fold changes from the differential expression results for IFNα-7 vs. IFNα-10 treatment. Default settings were used for the runGSA function in piano and the heatmap was made with the seaborn clustermap function. Numbers represent p values. Next page:


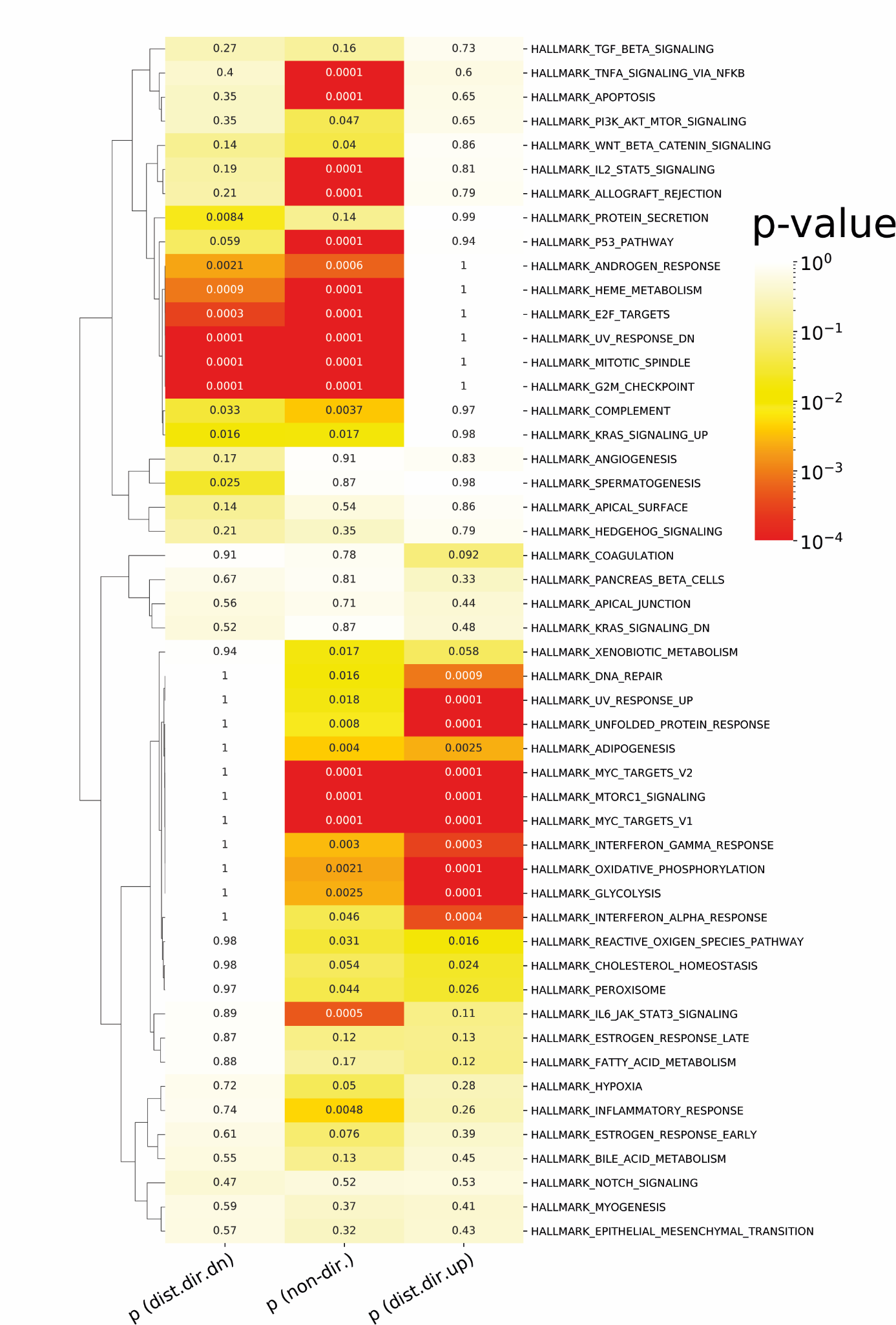


Fig. S3. Gene set variation analysis (GSVA) of gene sets annotated to be regulated by STAT transcription factors in the Treg cells at day 0 (control) or treated with IFNα-10, IFNα-7 or PBS.


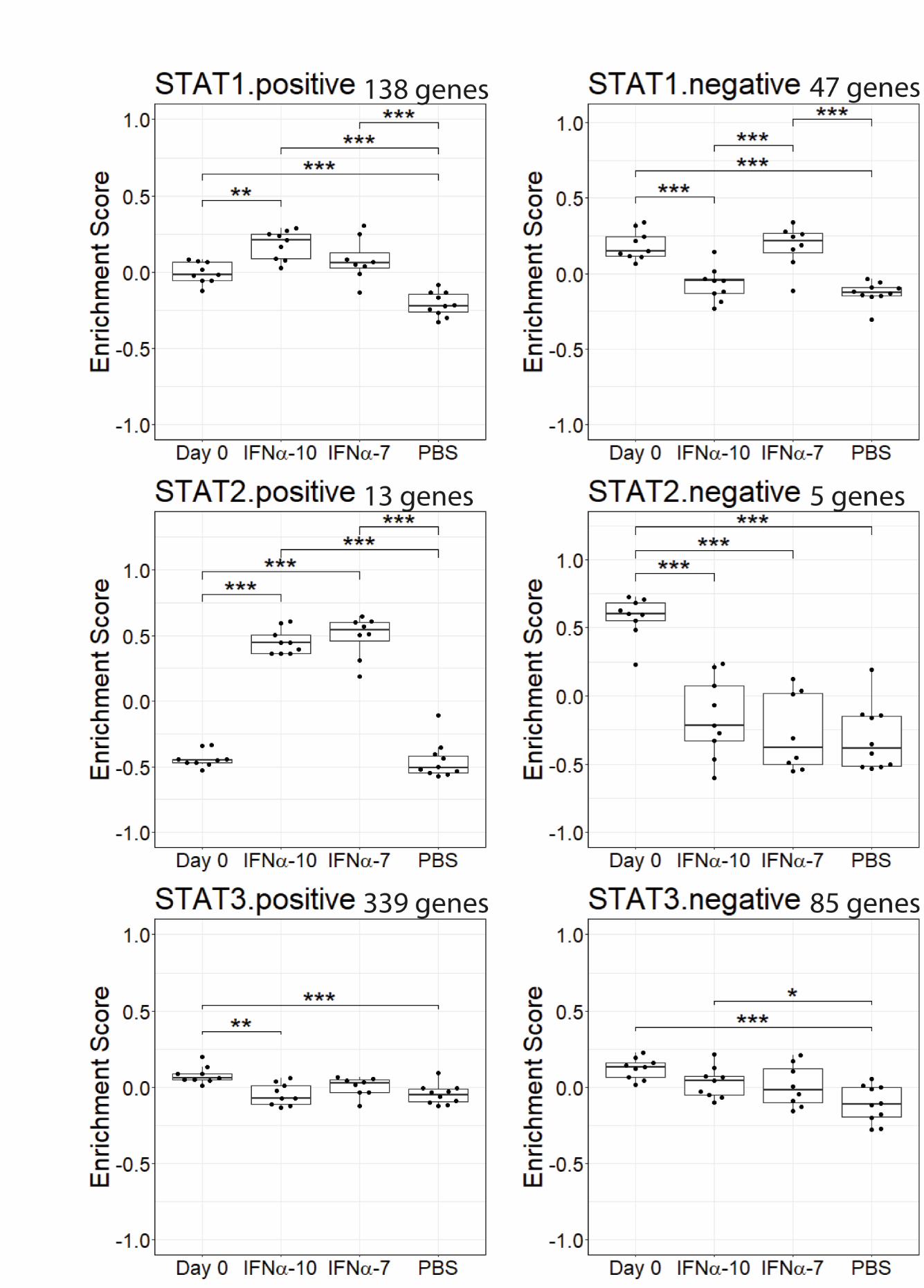


**
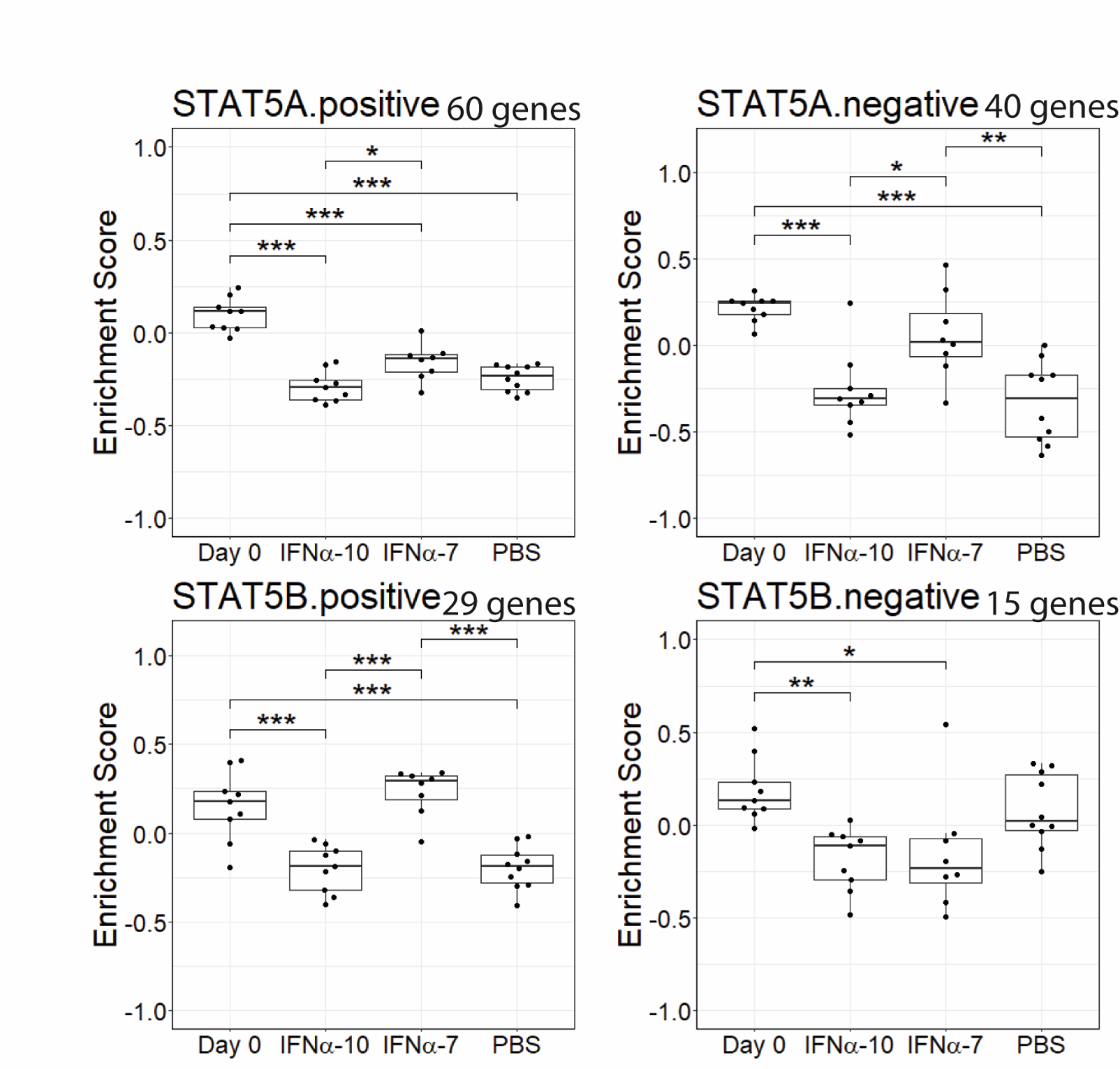
**

Fig. S4. TPM expression values for BMPR1A, BMPR1B, BMPR2 and ADORA2A (A2a), at day 0 (control) or treated with IFNα-10, IFNα-7 or PBS.


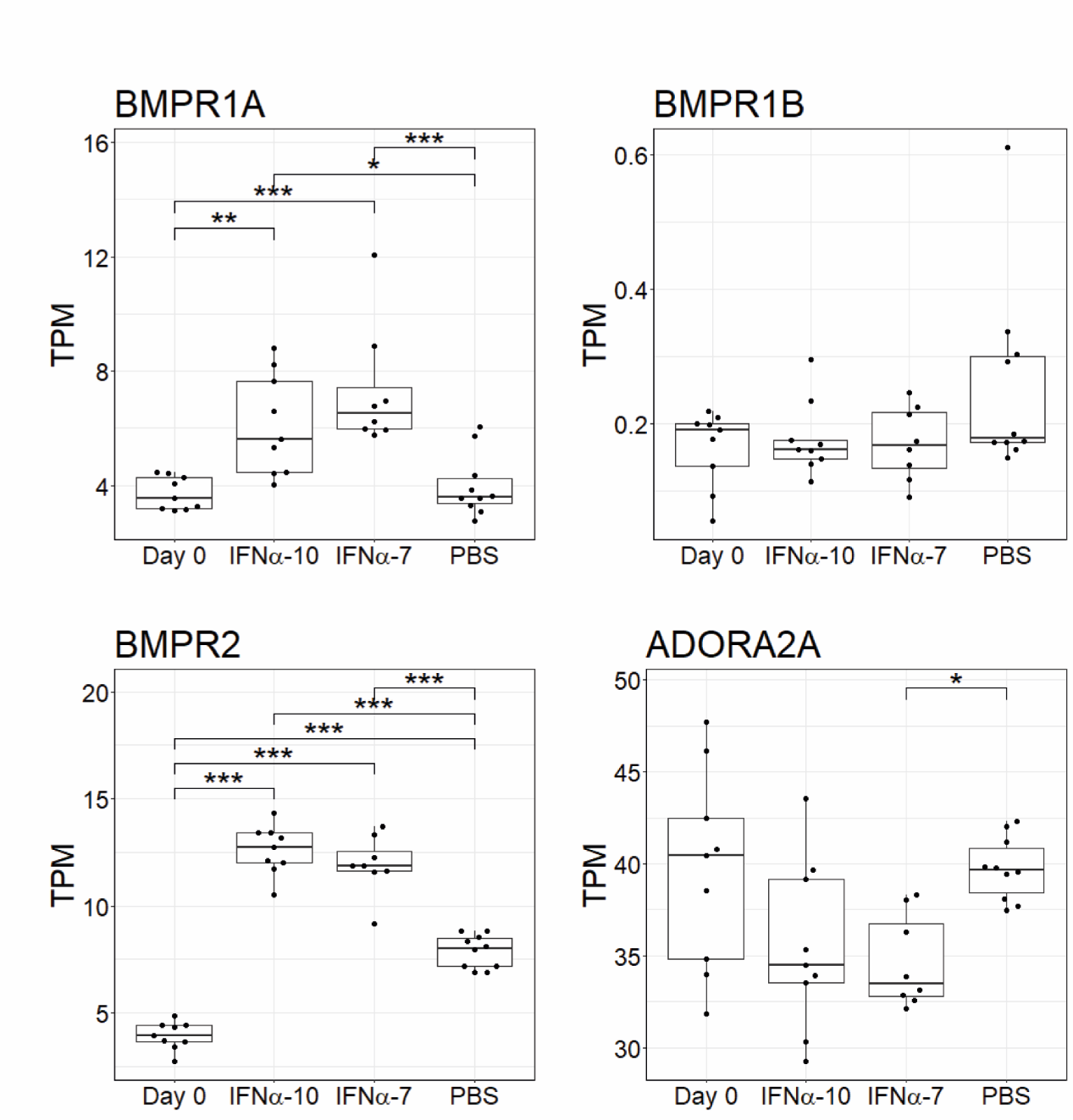


Fig. S5. TPM expression values for IFNΑR1, IFNΑR2, IFNGR1and IFNGR2, at day 0 (control) or treated with IFNα-10, IFNα-7 or PBS.


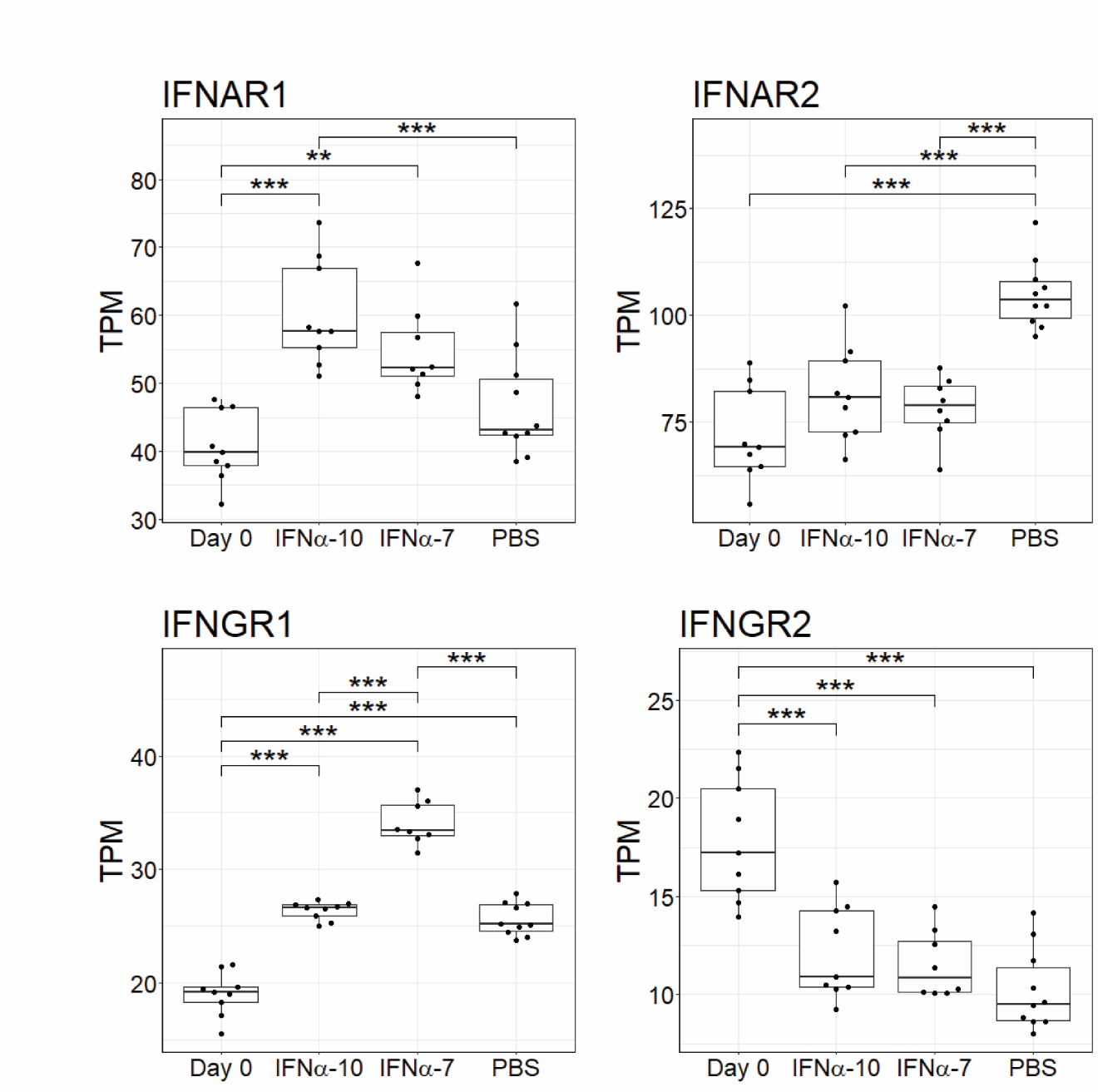


Fig. S6. Gating strategy for sorting naïve CD4^+^CD25^high^CD127^low^CD45RO^-^ Treg cells (A) and for gating live FOXP3^+^, CTLA4^+^ cells (B).


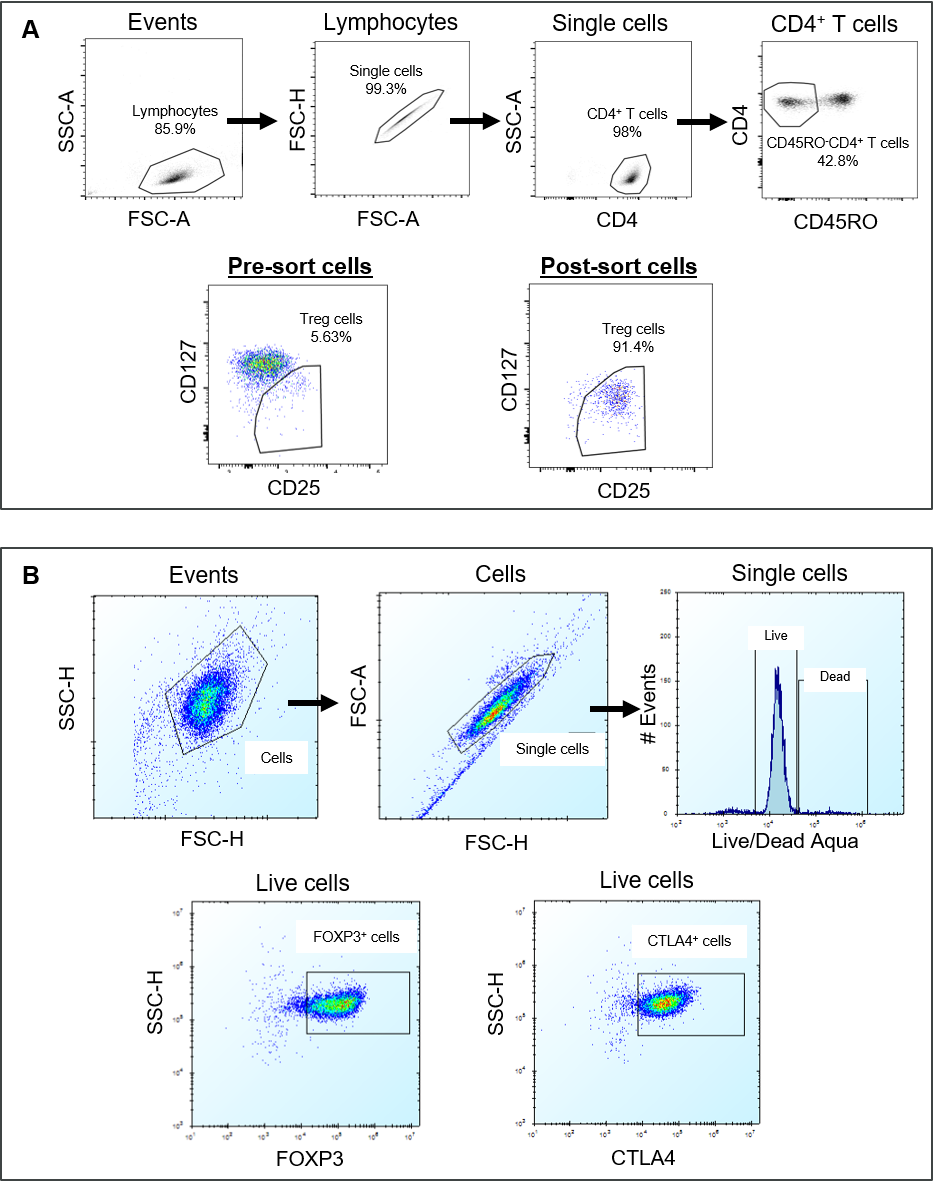


**Fig. S7. PBS neutral control treated cells from five different donors.** % FOXP3^+^ live cells (A), % CTLA4^+^ live cells (B), and cell viability (C). Cells from Donor E and F were used in the primary screening to screen 575 proteins at two concentrations. Cells from Donor 1, 2, 3 were used to follow up the hits identified from the primary screening in ten-point concentration response curve experiments. n=24 data points for Donor E and F; n=12-14 data points for Donor 1, 2, 3.





**Supplementary Tables**

**Table S1:** Excel-document. List of produced proteins tested in the Treg screen. Column A- Treg screen. Column B- Uniprot_id. Column C- Gene_name. Column D- Stock concentration (µM) of proteins screened in the primary assay.

**Table S2:** Excel-sheet listing differential gene expression analysis results based on the different RNAseq samples.
